# Efficacy of plant extracts in heart failure patients: a systematic review and network meta-analysis

**DOI:** 10.1186/s12872-026-05793-x

**Published:** 2026-06-12

**Authors:** Tianjiao Tang, Guang Li, Xiaoqian Huang, Hongsheng Liang

**Affiliations:** Department of Cardiology, Dongguan Children’s Hospital Affiliated to Guangdong Medical University, 68 South Xihu Third Road, Shilong, Dongguan China

**Keywords:** plant extracts, heart failure, network meta-analysis

## Abstract

**Objective:**

This study aimed to evaluate the therapeutic efficacy of plant extracts in patients with heart failure.

**Methods:**

A systematic search was conducted in PubMed, EMBASE, Cochrane Library, and Web of Science to identify randomized controlled trials (RCTs) investigating the effects of plant extracts on heart failure patients. The search period spanned from database inception to June 2024. The methodological quality of the included studies was assessed using the Cochrane Risk of Bias Tool. Statistical analyses were performed using Review Manager 5.3 and Stata 15.1.

**Results:**

A total of 28 studies involving 3,650 heart failure patients were included. Network meta-analysis examined 15 distinct plant extracts: Panax ginseng and Ophiopogon japonicus extract, Water extract of stem bark of Terminalia arjuna, Astragalus extract, Panax ginseng extract, Red ginseng extract, Astragalus and Codonopsis extract, Hawthorn extract, Red ginseng Ophiopogon japonicus and Schisandra extract, Ginkgo biloba extract, Salvia miltiorrhiza extract, Rhodiola extract, Panax ginseng and Aconite extract, Centaurea behen extract, Oak wood extract, and Berberine extract. Based on the Surface Under the Cumulative Ranking Curve (SUCRA) values, Water extract of stem bark of Terminalia arjunaranked first in Number of patients with improved New York Heart Association (NYHA) classification (80.4%). Salvia miltiorrhizaextract exhibited the highest efficacy in enhancing left ventricular ejection fraction (LVEF) (89.7%), while Oak wood extract demonstrated optimal improvement in quality of life (QoL) (100%). Astragalusextract showed superior efficacy in the six-minute walk test (6MWT) (89.5%).

**Conclusion:**

In summary, our study supports the therapeutic potential of plant extracts for heart failure. Based on SUCRA rankings, they can be effective adjunctive therapies to improve cardiac function, quality of life, and prognosis, with different extracts offering distinct advantages. However, the limited studies for some interventions and scarce direct comparative evidence necessitate cautious interpretation. Future work should prioritize larger, high-quality RCTs for validation.

**Trial registration:**

This meta-analysis was conducted in accordance with the Cochrane Handbook and the PROSPERO NMA checklist, and was registered on PROSPERO (Registration No: CRD42024609307).

**Supplementary Information:**

The online version contains supplementary material available at 10.1186/s12872-026-05793-x.

## Introduction

Heart Failure (HF) is a complex clinical syndrome caused by multiple etiological factors, characterized by structural and/or functional abnormalities of the heart that impair ventricular pumping capacity. Its predominant clinical manifestations include dyspnea, fatigue, and fluid retention (pulmonary congestion, systemic venous congestion, and peripheral edema) [1]. As a severe consequence or end-stage manifestation of various cardiovascular diseases, Heart failure is associated with persistently high mortality and rehospitalization rates. The global prevalence is estimated to exceed 64 million cases, imposing a substantial burden on public health. Impaired QoL, frequent hospitalizations, suboptimal disease management, population aging, and increasing risk factors are all closely associated with the pathogenesis and progression of heart failure [2, 3]. According to statistics, the prevalence of heart failure in Western countries ranges between 1% and 14%, showing an upward trend [4, 5]. In Asian countries such as China, the prevalence of heart failure has also been increasing annually, particularly among the elderly population [6]. Although the age-adjusted incidence of heart failure has declined globally, its prevalence continues to rise due to population aging trends [7]. The growing global burden of heart failure imposes substantial economic pressure on public health systems. Therefore, exploring effective treatment strategies is of paramount importance for improving the prognosis of heart failure patients.

Despite significant advancements in existing treatment plans for heart failure, challenges such as drug side effects, poor patient compliance, and limited long-term efficacy persist, presenting formidable obstacles in heart failure management. Consequently, adjunctive therapies for heart failure have emerged as a research focus [8]. As an alternative and complementary approach, plant extracts have garnered increasing attention in heart failure treatment in recent years. Given that heart failure is associated with multiple risk factors, multi-target therapeutic strategies may offer enhanced efficacy [9]. Plant extracts, characterized by their natural origin, mild toxicity profile, and multi-target therapeutic effects, represent promising candidates for heart failure treatment [10]. Research indicates that ginsenosides exert beneficial effects primarily by improving energy metabolism, suppressing excessive autophagy, exerting antioxidant and anti-inflammatory actions, and promoting exosome secretion, the AMPK and PPAR signaling pathways are likely pivotal targets underlying these diverse cardioprotective mechanisms [11]. Astragaloside IV has been shown to alleviate symptoms in heart failure patients through multiple mechanisms, including protection against myocardial ischemia, regulation of sarcoplasmic reticulum calcium pumps, promotion of angiogenesis, improvement of energy metabolism, inhibition of cardiac hypertrophy and fibrosis, and reduction of cardiac cell apoptosis [12]. Ginkgo biloba extracts demonstrate therapeutic potential in mitigating inflammation, improving myocardial function, and reducing myocardial injury [13]. The hawthorn extract WS1442 has been confirmed to safely enhance exercise capacity and alleviate symptoms such as chest tightness and dyspnea in heart failure patients, with effects exhibiting dose-dependency [14]. Tai’s reported that with proper administration, aconite extracts showed no significant difference in adverse event incidence compared to controls, while long-term use played an important role in preventing composite cardiovascular events [15]. However, systematic comparisons among different plant extracts remain lacking, and variations in their mechanisms of action, characteristics, and therapeutic efficacy warrant further investigation. This study aims to employ network meta-analysis to compare the effects of various plant extracts in heart failure patients, rank their efficacy, and provide evidence-based medical guidance for clinical practice.

Network meta-analysis (NMA) is a statistical methodology that synthesizes both direct and indirect evidence to compare the effects of multiple interventions while estimating the relative ranking of each treatment’s efficacy, thereby facilitating the selection of optimal treatment plans [16]. Accordingly, this study systematically compared the therapeutic efficacy of various botanical extracts in patients with heart failure and provided evidence-based recommendations to guide clinical decision-making for both patients and physicians.

## Materials and methods

This meta-analysis was conducted in accordance with the Cochrane Handbook and the PROSPERO NMA checklist, and was registered on PROSPERO (Registration No: CRD42024609307). The reporting of this systematic review followed the Preferred Reporting Items for Systematic Reviews and Meta-Analyses (PRISMA) 2020 statement. The completed PRISMA 2020 checklist is provided as Supplementary File 1.

### Search strategy

A comprehensive search was conducted across four electronic databases (PubMed, EMBASE, the Cochrane Library, and Web of Science) from their inception to June 2024. The search strategy was developed based on the PICOS framework: (P) Population: Patients with heart failure; (I) Intervention: Plant extracts; (C) Comparator: Conventional treatments, including Western medications; (O) Outcomes: Number of patients with improved NYHA classification, LVEF, 6MWT, and QoL; (S) Study design: RCTs. The detailed search strategy is provided in Supplementary Table S1, S2, S3, and S4. It should be noted that this search was restricted to published literature. Therefore, citation tracking was not performed, and clinical trial registries such as ClinicalTrials.gov or the Chinese Clinical Trial Registry (ChiCTR) were not searched.

### Inclusion criteria

(1) Study design: All included studies were RCTs; (2) Participants: All patients aged ≥ 18 years diagnosed with heart failure who meeting the diagnostic criteria and classified as NYHA functional class II–IV (NYHA classification was based on the 2022 AHA/ACC/HFSA Heart Failure Guideline, defining classes I-IV as: Class I, no limitation; Class II, slight limitation with ordinary activity; Class III, marked limitation with less-than-ordinary activity; Class IV, symptoms at rest.); (3) Interventions: The experimental group received conventional Western medicine (including diuretics, angiotensin-converting enzyme inhibitors [ACEIs] or angiotensin receptor blockers [ARBs], digitalis preparations, β-blockers, aldosterone receptor antagonists, nitrates, etc.) in accordance with contemporary heart failure treatment guidelines [1], supplemented with plant extracts. The control group received only conventional Western medicine; (4) Outcome measures: At least one of the following was assessed: Number of patients with improved NYHA classification, LVEF, 6MWT, or QoL.

### Exclusion criteria

(1) Duplicate publications; (2) Nonclinical randomized controlled trials; (3) Literature with incomplete data or inability to obtain full text; (4) Guidelines, reviews, conferences, meta-analyses, animal and cell experiments.

### Study selection

The reference management software EndNote X 20.0 was used exclusively to remove duplicate references and filter out unwanted references. Two authors first screened the titles of replicates, nonrandomized controlled trials, review papers, conference papers, and meta-analyses. Two authors screened abstracts for exclusion criteria. The remaining articles were independently assessed by two authors for inclusion eligibility. Publications were included only if both authors agreed on their inclusion. We used double entry and cross- checking in the process of data extraction in order to avoid data errors. Any disagreements were resolved through discussion or by consulting a third reviewer.

### Data extraction

Standardized and preselected data extraction tables were used to record data to be included in studies under the following headings: (1) author, (2) country, (3) year of publication, (4) number of study participants, (5) patient mean age, (6) total sample size, (7) intervention, and (8) study period.

### Risk of bias

Two researchers independently assessed the risk of bias (ROB) in accordance with the Cochrane Handbook version 5.4.0 tool for assessing ROB in RCTs. The following seven domains were considered: (1) randomized sequence generation, (2) treatment allocation concealment, blinding of (3) participants and (4) personnel, (5) incomplete outcome data, (6) selective reporting, and (7) other sources of bias. Trials were categorized into three levels of ROB by the number of components for which high ROB potentially existed: high risk (five or more), moderate risk (three or four), and low risk (two or fewer) [17].

### Data analysis

Statistical analysis using Stata 15.1 and Review Manager 5.3. According to the characteristics of the data type, the effect size that can reasonably reflect the overall data was selected. If the analysis data were continuous data, the effect size was the mean difference (MD). The odds ratio (OR) was used if the data were binary. Effect sizes were presented with 95% confidence intervals (CI).

We used Stata 15.1 and based on the PRISMA NMA instruction manual, to simulate chains using Markov chain Monte Carlo simulation chains in a Bayesian-based framework for NMA aggregation and analysis [18]. We use the node method to quantify and prove the agreement between indirect and direct comparisons, calculated by instructions in the Stata software, and if the p‐value > 0.05, the consistency is verified [19]. The Stata software was employed to analyze and describe the therapeutic effects and differential actions of various plant extracts in the network meta-analysis of heart failure treatment. The intervention network map is an intuitive representation of the evidence base, where each node in the generated network map represents a different intervention and different control conditions, and the lines connecting the nodes represent direct head-to-head comparisons between interventions. The size of each node and the width of the connecting line are proportional to the number of studies [20].

The probability values were summarized and reported as the area under the cumulative ranked SUCRA curve, which was 0 when the treatment effect was the worst and 1 when the treatment effect was the best; the greater the area under the SUCRA curve, the better the treatment effect [20, 21]. While the P score or SUCRA can be usefully re-expressed as the percentage of effectiveness or acceptability of the plant extracts, such scores should be interpreted cautiously unless there are actual clinically meaningful differences between interventions [22]. To check for the presence of bias due to small-scale studies, which may lead to publication bias in NMA, a network funnel plot was generated and visually inspected using the criterion of symmetry [23].

## Results

### Study selection

**Fig. 1:**
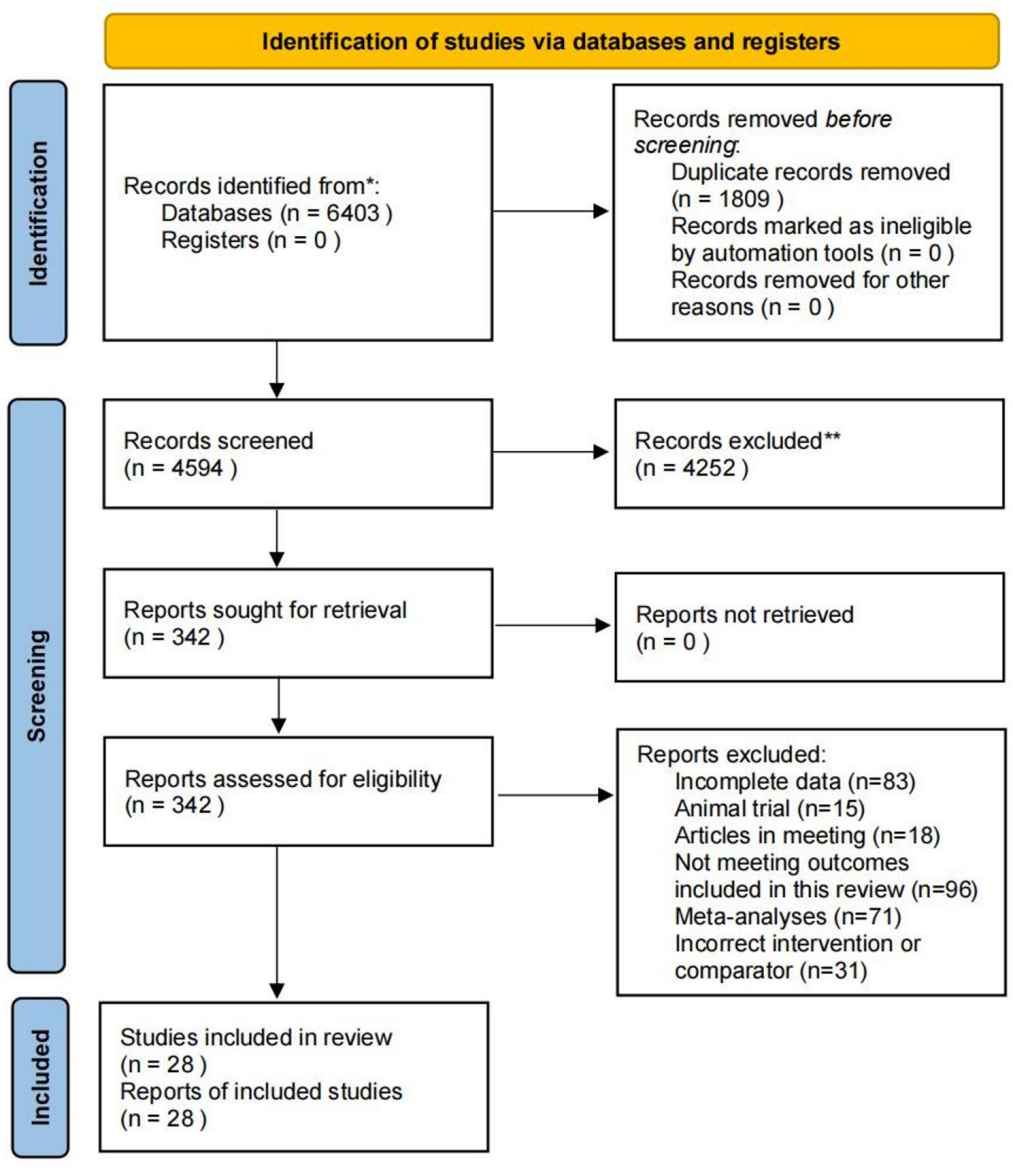
Flow diagram of literature selection

The initial research resulted in 6403 references. After removing duplicates, reading the titles and abstracts of the remaining 4594 references, 4252 references were excluded. Searching the remaining 342 full text references, 314 did not meet the eligibility criteria (reasons for exclusion included incomplete data, animal experiments, conference proceedings, failure to meet the results included in this study, incorrect interventions or comparisons, meta-analyses). The remaining 28 studies were included in the meta-analysis (Fig. 1).

### Risk of bias assessment

The risk of bias for included studies was evaluated with the Cochrane Risk of Bias Tool. The included studies described random sequence generation, had no incomplete data, did not report selectively, and were assessed as low risk. Three studies showed allocation concealment and a low risk of bias. Twenty-four studies were blinded to assessors and were classified as at low risk of detection bias. Two studies showed additional bias due to small sample sizes and a high risk of bias. Detailed information is provided in Supplementary Figure S1.

### Characteristics of the included studies

A total of 28 RCTs involving 3,650 patients with heart failure were included. The mean age of the included participants was 61.4 ± 8.6 years, corresponding to an estimated 95% reference interval of approximately 44.5 to 78.3 years based on the assumption of a normal distribution. Fifteen herbal extracts were compared, including Astragalus extract (6 studies) [24–29], Hawthorn extract (4 studies) [30–33], Panax ginseng and Ophiopogon japonicus extract (4 studies) [34–37], Berberine extract (2 studies) [38, 39], Water extract of stem bark of Terminalia arjuna (2 studies) [40, 41], Panax ginseng extract (1 study) [42], Centaurea behen extract (1 study) [43], Astragalus and Codonopsis extract (1 study) [44], Oak wood extract (1 study) [45], Red ginseng extract (1 study) [46], Red ginseng Ophiopogon japonicus and Schisandra extract (1 study) [47], Ginkgo biloba extract (1 study) [48], Salvia miltiorrhiza extract (1 study) [49], Rhodiola extract (1 study) [50], and Panax ginseng and Aconite extract (1 study) [51]. The primary outcomes assessed included Number of patients with improved NYHA classification, LVEF, 6MWT, or QoL. The characteristics of the included studies are summarized in Table S1.

### Network meta-analysis

#### Network meta-analysis of the efficacy of number of patients with improved NYHA classification

All P-values for indirect and direct comparisons between all studies were tested for consistency and inconsistency, and p‐values were greater than 0.05, indicating that the effect of consistency between studies was acceptable. Details will be shown in Supplementary Table S5.

**Fig. 2:**
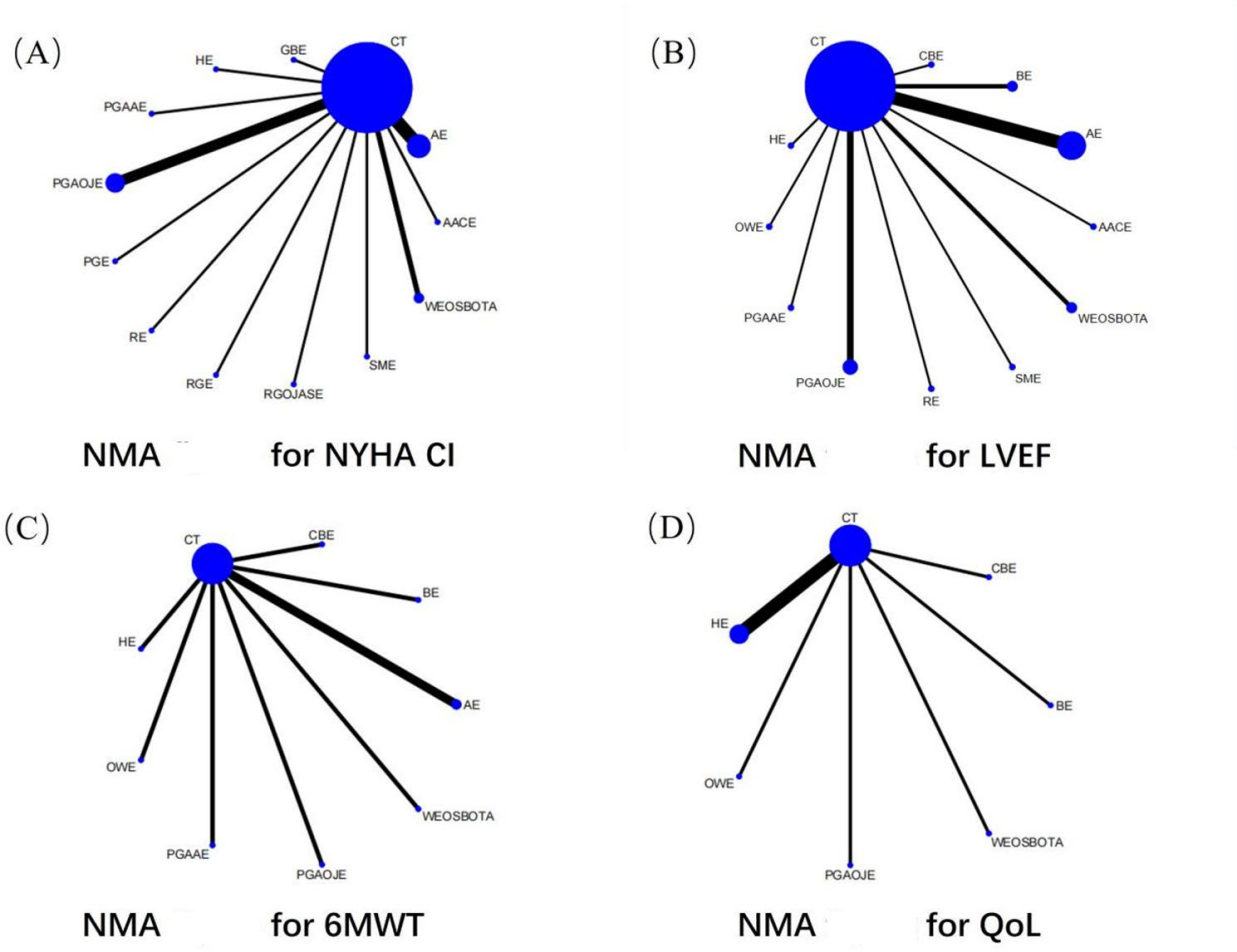
NMA figure for each outcome indicator. NYHA CI, LVEF, 6MWT, QoL NOTE: AE: Astragalus extract, AACE: Astragalus and codonopsis extract, BE: Berberine extract, CBE: Centaurea behen extract, CT: Conventional treatment, GBE: Ginkgo biloba extract, HE: Hawthorn extract, OWE: Oak wood extract PGAAE: Panax ginseng and Aconite extract, PGAOJE: Panax ginseng and Ophiopogon japonicus extract, PGE: Panax ginseng extract, RE: Rhodiola extract, RGE: Red ginseng extract, RGOJASE: Red ginseng Ophiopogon japonicus and Schisandra extract, SME: Salvia miltiorrhiza extract, WEOSBOTA: Water extract of stem bark of Terminalia arjuna, NYHA CI: Number of patients with improved New York Heart Association classification, LVEF: Left Ventricular Ejection Fraction 6MWT: 6-Minute Walk Test, QoL: Quality of Life

**Fig. 3:**
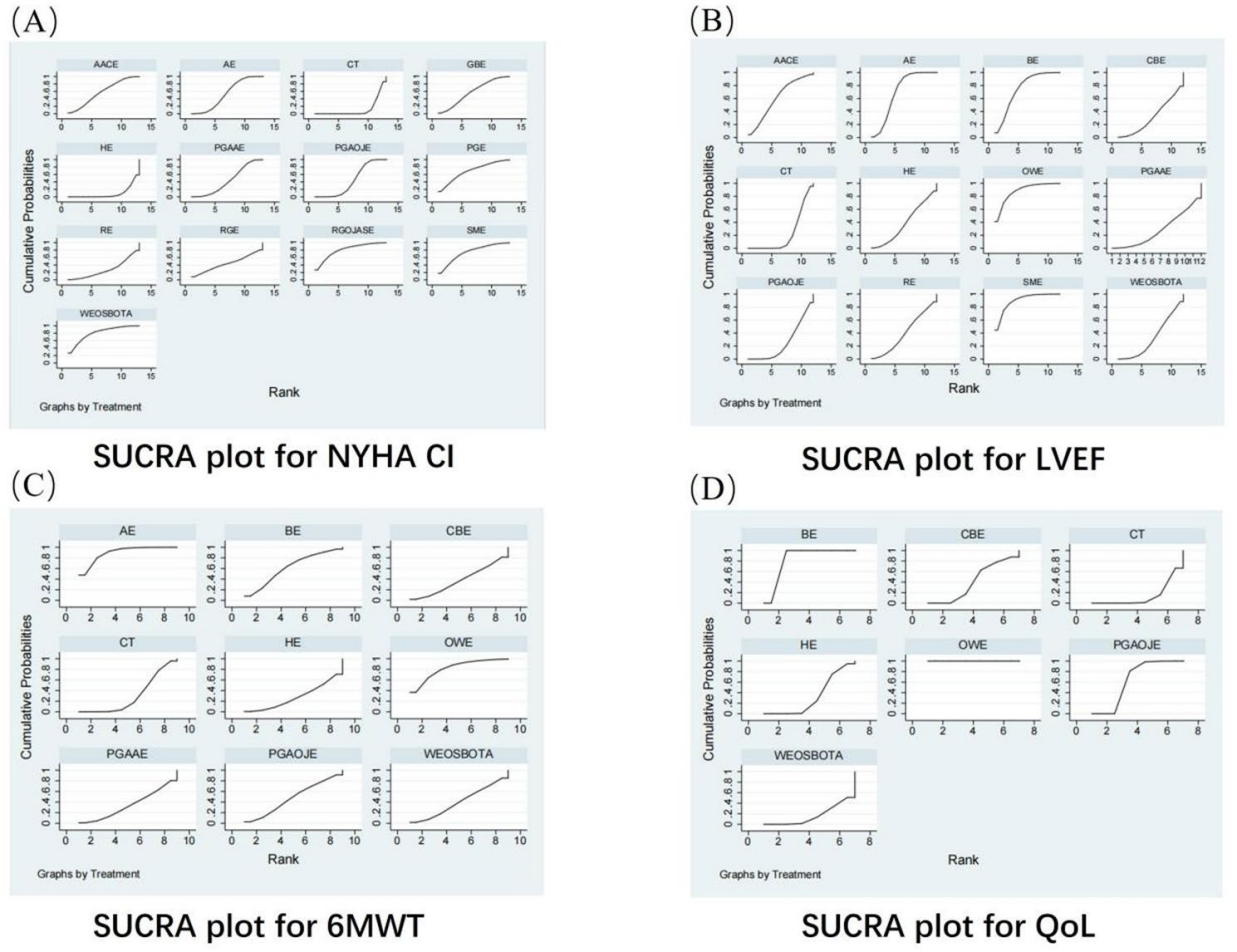
SUCRA values for each outcome indicator. NYHA CI, LVEF, 6MWT, QoL NOTE: AE: Astragalus extract, AACE: Astragalus and codonopsis extract, BE: Berberine extract, CBE: Centaurea behen extract, CT: Conventional treatment, GBE: Ginkgo biloba extract, HE: Hawthorn extract, OWE: Oak wood extract PGAAE: Panax ginseng and Aconite extract, PGAOJE: Panax ginseng and Ophiopogon japonicus extract, PGE: Panax ginseng extract, RE: Rhodiola extract, RGE: Red ginseng extract, RGOJASE: Red ginseng Ophiopogon japonicus and Schisandra extract, SME: Salvia miltiorrhiza extract, WEOSBOTA: Water extract of stem bark of Terminalia arjuna, NYHA CI: Number of patients with improved New York Heart Association classification, LVEF: Left Ventricular Ejection Fraction 6MWT: 6-Minute Walk Test, QoL: Quality of Life

Twenty studies involving 13 interventions were included for Number of patients with improved NYHA classification, with the network evidence diagram presented in Fig. 2A. The network meta-analysis demonstrated that the following interventions significantly outperformed the control group in Number of patients with improved NYHA classification: Water extract of stem bark of Terminalia arjuna [MD = 6.89, 95% CI=(1.39, 34.26)], Red ginseng Ophiopogon japonicus and Schisandra extract [MD = 7.00, 95% CI=(1.38, 35.48)], Salvia miltiorrhiza extract [MD = 5.52, 95% CI=(1.11, 27.43)], Astragalus and Codonopsis extract [MD = 3.14, 95% CI=(1.07, 9.27)], Ginkgo biloba extract [MD = 3.14, 95% CI=(1.06, 9.27)], Astragalus extract [MD = 2.74, 95% CI=(1.66, 4.54)], Panax ginseng and Aconite extract [MD = 2.28, 95% CI=(1.09, 4.74)], Panax ginseng and Ophiopogon japonicus extract [MD = 2.31, 95% CI=(1.84, 2.91)], detailed results are presented in Supplementary Table S6. Cumulative ranking probability plot was generated through statistical analysis of the 20 interventions, with corresponding SUCRA values calculated to rank treatment plans. The SUCRA curve area diagram (Fig. 3A), generated using Stata 15.1, yielded the following efficacy ranking: Water extract of stem bark of Terminalia arjuna (80.4%) > Red ginseng Ophiopogon japonicus and Schisandra extract (80.2%) > Salvia miltiorrhiza extract (74.0%) > Panax ginseng extract (68.7%) > Astragalus and Codonopsis extract (57.6%) > Ginkgo biloba extract (57.5%) > Astragalus extract (52.8%) > Red ginseng extract (43.7%) > Panax ginseng and Aconite extract (43.6%) > Panax ginseng and Ophiopogon japonicus extract (43.2%) > Rhodiola extract (27.7%) > Conventional treatment (11.8%) > Hawthorn extract (8.9%). Based on the SUCRA curve area analysis, Water extract of stem bark of Terminalia arjuna demonstrated the highest ranking for Number of patients with improved NYHA classification.

#### Network meta-analysis of the efficacy of LVEF

All P-values for indirect and direct comparisons between all studies were tested for consistency and inconsistency, and p‐values were greater than 0.05, indicating that the effect of consistency between studies was acceptable. Details will be shown in Supplementary Table S7.

A total of 20 studies involving 12 interventions were included, and the network evidence diagram is presented in Fig. 2B. The network meta-analysis results demonstrated that, compared with the control group (conventional treatment), the following interventions showed superior efficacy in improving LVEF: Salvia miltiorrhiza extract [MD = 1.62, 95% CI = (0.49, 2.75)], Oak wood extract [MD = 1.58, 95% CI = (0.34, 2.82)], Berberine extract [MD = 1.05, 95% CI = (0.21, 1.89)], Astragalus extract [MD = 0.89, 95% CI = (0.42, 1.35)], detailed information is provided in Supplementary Table S8. A statistical analysis of the 20 interventions was conducted to generate a cumulative ranking probability plot, and the corresponding SUCRA values were calculated to rank the treatment plans. Based on the SUCRA curve area diagram (Fig. 3B) generated by Stata 15.1, the ranking was as follows: Salvia miltiorrhiza extract (89.7%) > Oak wood extract (87.7%) > Berberine extract (74.6%) > Astragalus extract (69.4%) > Astragalus and Codonopsis extract (60.7%) > Rhodiola extract (39.7%) > Hawthorn extract (38.4%) > Water extract of stem bark of Terminalia arjuna (32.7%) > Centaurea behen extract (31.3%) > Panax ginseng and Aconite extract (28.3%) > Panax ginseng and Ophiopogon japonicus extract (25.5%) > Conventional treatment (21.9%). The SUCRA curve area diagram indicated that Salvia miltiorrhiza extract ranked first in improving LVEF.

#### Network meta-analysis of the efficacy of 6MWT

All P-values for indirect and direct comparisons between all studies were tested for consistency and inconsistency, and p‐values were greater than 0.05, indicating that the effect of consistency between studies was acceptable. Details will be shown in Supplementary Table S9.

A total of nine studies involving nine interventions were included for 6MWT, with the network evidence diagram presented in Fig. 2C. The network meta-analysis demonstrated that Astragalus extract [MD = 1.09, 95%CI=(0.34,1.85)] yielded superior outcomes in 6MWT compared to conventional treatment in the control group, with detailed results shown in Supplementary Table S10. Statistical analysis of the nine interventions generated cumulative ranking probability plots, with treatment plan rankings determined by corresponding SUCRA values. The SUCRA curve area diagram (Fig. 3C), generated using Stata 15.1 revealed the following efficacy ranking: Astragalus extract (89.5%) > Oak wood extract (81.8%) > Berberine extract (61.3%) > Panax ginseng and Ophiopogon japonicus extract (47.6%) > Water extract of stem bark of Terminalia arjuna (40.2%) > Centaurea behen extract (37.4%) > Panax ginseng and Aconite extract (34.2%) > Conventional treatment (30.2%) > Hawthorn extract (27.47%). Based on the SUCRA curve area ranking, Astragalus extract demonstrated the highest efficacy among all interventions for 6MWT outcomes.

#### Network meta-analysis of the efficacy of QoL

All P-values for indirect and direct comparisons between all studies were tested for consistency and inconsistency, and p‐values were greater than 0.05, indicating that the effect of consistency between studies was acceptable. Details will be shown in Supplementary Table S11.

Regarding QoL, a total of 9 studies involving 7 substances were included, with the network evidence map presented in Fig. 2D. The network meta-analysis results demonstrated that, in terms of improving QoL, Oak wood extract [MD = 16.44, 95% CI = (12.55, 20.34)], Berberine extract [MD = 5.29, 95% CI = (4.59, 6.00)], and Panax ginseng and Ophiopogon japonicus extract [MD = 0.58, 95% CI = (0.23, 0.93)] were superior to the control group (conventional treatment), with detailed data shown in Supplementary Table S12. Through statistical analysis of the 9 substances, a cumulative ranking probability plot was generated, and the corresponding SUCRA values were calculated to rank the treatment plans. The SUCRA curve area plot (Fig. 3D), generated using Stata 15.1 software, yielded the following ranking: Oak wood extract (100%) > Berberine extract (83.3%) > Panax ginseng and Ophiopogon japonicus extract (63.2%) > Centaurea behen extract (40.9%) > Hawthorn extract (32.5%) > Water extract of stem bark of Terminalia arjuna (16.0%) > Conventional treatment (13.9%). Based on the SUCRA curve area ranking, Oak wood extract was ranked first in improving quality of life.

### Publication bias test

**Fig. 4:**
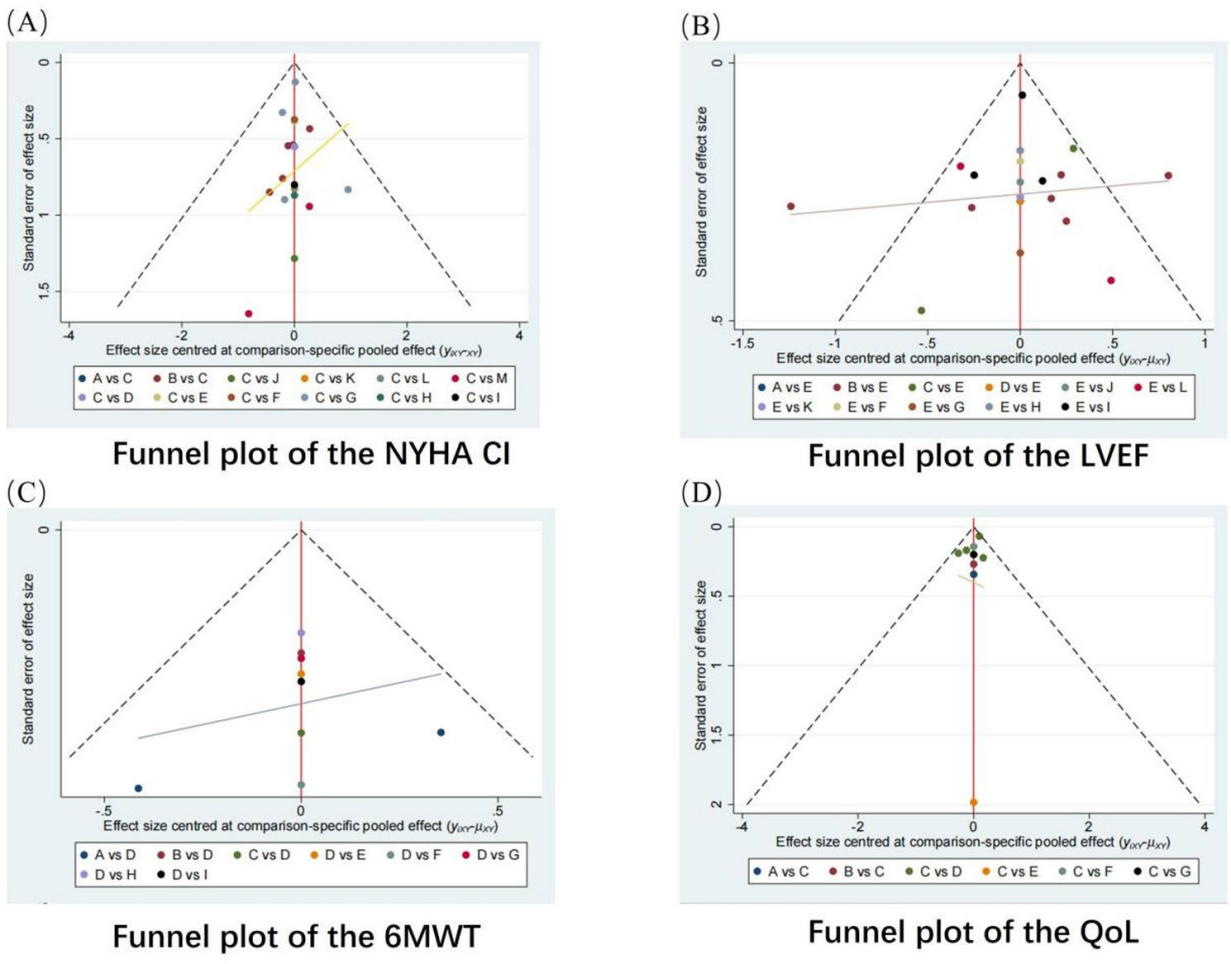
Funnel plots for each outcome indicator. NYHA CI, LVEF, 6MWT, QoL **A** NYHA CI: A: Astragalus and codonopsis extract; B: Astragalus extract; C: Conventional treatment; D: Ginkgo biloba extract; E: Hawthorn extract; F: Panax ginseng and Aconite extract; G: Panax ginseng and Ophiopogon japonicus extract; H: Panax ginseng extract; I: Rhodiola extract; J: Red ginseng extract; K: Red ginseng, Ophiopogon japonicus and Schisandra extract; L: Salvia miltiorrhiza extract; M: Water extract of stem bark of Terminalia arjuna **B** LVEF: A: Astragalus and codonopsis extract; B: Astragalus extract; C: Berberine extract; D: Centaurea behen extract; E: Conventional treatment; F: Hawthorn extract; G: Oak wood extract; H: Panax ginseng and Aconite extract; I:Panax ginseng and Ophiopogon japonicus extract; J: Rhodiola extract; K: Salvia miltiorrhiza extract; L: Water extract of stem bark of Terminalia arjuna **C** 6MWT: A: Astragalus extract; B: Berberine extract; C: Centaurea behen extract; D: Conventional treatment; E: Hawthorn extract; F: Oak wood extract; G: Panax ginseng and Aconite extract; H: Panax ginseng and Ophiopogon japonicus extract; I: Water extract of stem bark of Terminalia arjuna **D** QoL: A: Berberine extract; B: Centaurea behen extract; C: Conventional treatment; D: Hawthorn extract; E: Oakwood extract; F: Panax ginseng and Ophiopogon japonicus extract; H: Water extract of stem bark of Terminalia arjuna

We constructed separate funnel plots for all outcome measures to test for possible publication bias. From the funnel plot, we can see that the points on both sides of the line are basically symmetrical, and we did not find any significant publication bias. Funnel plots of Number of patients with improved NYHA classification, LVEF, 6MWT, and QoL are shown in Fig. 4.

## Discussion

This study compared the therapeutic efficacy of various plant extracts in combination with conventional therapypatients with heart failure. A total of 28 studies involving 15 distinct plant extracts were included, encompassing 3,650 heart failure patients, representing a substantial sample size. Our findings demonstrate that, compared with conventional therapy alone, combination therapy with plant extracts exhibits superior efficacy in improving both LVEF and QoL. Among these, the add-on use of Salvia miltiorrhiza extract showed the most pronounced effect in enhancing LVEF, whereas the add-on use of Oak wood extract demonstrated optimal performance in improving QoL. Regarding Number of patients with improved NYHA classification, the add-on use of Water extract of stem bark of Terminalia arjuna yielded the most favorable outcomes, while the add-on use of Astragalus extract showed the greatest efficacy in 6MWT.

The NYHA classification is closely associated with prognostic indicators such as hospitalization and mortality rates in patients with heart failure [52, 53]. Since the original literature primarily reported the proportion of patients achieving improvement in NYHA class or overall trends of change without quantifying specific grade improvements, this study employed the Number of patients with improved NYHA classification. A current limitation of this research is the lack of data on specific numerical changes in NYHA classification. It is recommended that future studies provide detailed reporting of such specific grade transition information. In this study, the add-on use of Water extract of stem bark of Terminalia arjuna demonstrated the most significant improvement in number of patients with improved NYHA classification. Terminalia arjuna is a commonly used traditional Chinese medicinal herb, and its Water extract of stem bark primarily contains various flavonoids, phenolic acids, and terpenoids. Among these, flavonoids and phenolic acids exhibit potent antioxidant effects, capable of scavenging free radicals [54]and mitigating oxidative stress-induced damage to cardiac cells, thereby preserving cardiac function [55]. Furthermore, Water extract of stem bark of Terminalia arjuna may enhance myocardial cell survival by modulating energy metabolism within cardiomyocytes [56], ultimately improving cardiac function. These pharmacological properties may collectively contribute to its potential therapeutic effects observed in our analysis, suggesting it as a promising candidate for heart failure management. However, it must be noted that none of the included original studies conducted stratified analysis or adjustment for patient comorbidities, the improvement in NYHA functional classification observed in this study may not be entirely attributable to the therapeutic effect of the plant extract, but could be partially influenced by differences in patients’ underlying comorbidities. This potential confounding factor should be taken into consideration when interpreting the results.

In a recent network meta-analysis on Salvia miltiorrhiza extract [57], various Salvia miltiorrhiza injections demonstrated therapeutic efficacy for heart failure without increasing the risk of adverse reactions when compared with conventional treatments. Studies have shown that approximately half of heart failure patients exhibit reduced LVEF [58]. In the present study, the add-on use of Salvia miltiorrhiza extract showed notable effectiveness in improving LVEF, which may be attributed to the following mechanisms: The active components of Salvia miltiorrhiza possess potent antioxidant properties that mitigate oxidative stress in cardiac cells and protect them from free radical damage [59]. Additionally, these components suppress inflammatory responses and reduce inflammatory cell infiltration in myocardial tissue [60].

Regarding ventricular remodeling, Salvia miltiorrhiza extract may inhibit the activity of fibrotic factors such as transforming growth factor-β (TGF-β), thereby reducing collagen deposition in cardiac cells and subsequently delaying myocardial fibrosis progression while improving ventricular function [61]. Consequently, Salvia miltiorrhiza extracts exert therapeutic effects in heart failure patients while maintaining a favorable safety profile, consistent with previous research findings [62].

The 6MWT is a commonly used indicator for assessing exercise tolerance in patients with heart failure. A meta-analysis on Astragalus extract demonstrated that, compared with conventional therapy, Astragalus extract and its combination therapy were more effective in improving the total clinical response rate and alleviating heart failure symptoms [63], which is consistent with the 6MWT results observed in the present study. This effect may be attributed to the inhibition of cardiac hypertrophy through suppression of calcium ion-mediated calcineurin/NFATc3 and CaMKII signaling cascades [64], as well as the attenuation of cardiac inflammatory responses via the TLR4/MyD88/NF-κB signaling pathway, thereby reducing myocardial inflammatory cell infiltration [65].

Improvement of QoL is a pivotal therapeutic objective in heart failure management, as it serves as a robust predictor for reduced hospitalization and mortality rates [66]. A published meta-analysis demonstrated that oral administration of Chinese herbal medicine combined with conventional therapy significantly enhances QoL in heart failure patients [67]. Among the studies included in our analysis, the add-on use of Oak wood extract exhibited pronounced therapeutic advantages, which may be attributed to its capacity to upregulate mitochondrial enzyme NADH-dehydrogenase expression, enhance ATP production efficiency, and promote skeletal muscle growth and differentiation through stimulation of protein synthesis, thereby increasing muscle mass [68, 69].

In this study, the majority of included literature primarily focused on efficacy outcomes, while reports on potential adverse events were either highly abbreviated or unsystematic. The reported adverse events associated with plant extracts were predominantly mild to moderate in severity, with a low incidence of serious adverse events. However, significant heterogeneity was observed across different studies. Although pooled data suggested that these plant extracts were relatively safe under trial conditions, the current evidence has notable limitations. Many included studies lacked systematic and comprehensive monitoring and reporting of adverse events. The observation periods were relatively short, making it difficult to assess long-term safety risks. Additionally, the sample sizes may have been insufficient to detect rare adverse events. It is recommended that future studies systematically and comprehensively collect and report safety data, as this is an indispensable component in evaluating the clinical value of plant extracts therapies.

In summary, our study demonstrates significant clinical implications. The Water extract of stem bark of Terminalia arjuna, Salvia miltiorrhiza extract, Astragalus extract, and oak wood extract exhibit notable therapeutic effects on heart failure across various aspects. Based on our findings, plant extracts may serve as an adjunctive treatment to improve cardiac function and quality of life in patients. Furthermore, these results provide a theoretical foundation for future clinical trials.

## Strengths and limitations

Our study represents the first network meta-analysis comparing the efficacy of different plant extracts in patients with heart failure. This investigation included 28 studies involving 3,650 heart failure patients, featuring a relatively large sample size. We systematically incorporated as many eligible studies as possible to compare the therapeutic effects of various plant extracts, thereby providing updated and more comprehensive evidence-based recommendations.

However, both our study and the included trials present several limitations. First, the search was limited to published RCTs in major databases; citation tracking and searches of clinical trial registries (e.g., ClinicalTrials.gov, ChiCTR) were not conducted. While our comprehensive search across multiple databases aimed to maximize coverage, this approach may have missed unpublished studies or ongoing trials, potentially introducing reporting bias. Second, due to the extensive variety of plant extracts, in-depth analysis could not be conducted for each specific extract, and the lack of relevant RCTs may have influenced the outcomes. Third, heterogeneity across all RCTs—including variations in patient populations, baseline clinical parameters, drug dosages, and treatment durations—might have affected the results. Fourth, the original studies provided insufficient reporting on patient comorbidities and did not conduct controlled analyses, which may introduce confounding bias. Fifth, the included studies inadequately reported adverse events, preventing this study from assessing the safety of plant extracts. Finally, the limited number of included studies may affect statistical power, and the scarcity of studies on certain interventions along with the lack of direct comparative evidence necessitates cautious interpretation of the current findings. Future research should prioritize conducting larger-scale, high-quality RCTs to validate and supplement the present conclusions.

## Conclusions

Based on the currently limited evidence, this network meta-analysis found that compared with conventional therapy alone, the add-on use of certain plant extracts is associated with improvements in multiple clinical indicators in patients with heart failure, suggesting the potential adjunctive therapeutic value of such combination regimens. Specifically, the add-on use of Salvia miltiorrhiza extract demonstrated superior efficacy in improving LVEF, while the add-on use of Oak wood extract exhibited significant advantages in enhancing QoL. The add-on use of Water extract of stem bark of Terminalia arjuna showed the highest Number of patients with improved NYHA classification, and the add-on use of Astragalus extract displayed remarkable improvement in 6MWT.

## Supplementary Information

Supplementary Material 1. Tables S1-S12.

Supplementary Material 2. Figure S1.

Supplementary Material 3. File 1.

## Data Availability

The data that support the findings of the study are available from the first author, upon reasonable request.
