# Supplementary material for: Efficacy of plant extracts in heart failure patients: a systematic review and network meta-analysis
Source: BMC Cardiovasc Disord. 2026 Jun 12;26:502. doi: 10.1186/s12872-026-05793-x (PMC13262503; doi:10.1186/s12872-026-05793-x)
Supplement: Supplementary file 1 — Supplementary Material 1. Tables S1-S12. [file 12872_2026_5793_MOESM1_ESM.pdf]

Supplementary Table S1. Search strategy on PubMed

#1	((Heart Failure[MeSH Terms]) OR (Heart Failure, Diastolic[MeSH Terms])) OR (Heart Failure, Systolic[MeSH Terms])
#2	((((((((((((Heart Failure[Title/Abstract]) OR (Heart Failure, Diastolic[Title/Abstract])) OR (Heart Failure, Systolic[Title/Abstract])) OR (Cardiac Failure[Title/Abstract])) OR (Heart Decompensation[Title/Abstract])) OR (Heart Failure, Congestive[Title/Abstract])) OR (Heart Failure, Right-Sided[Title/Abstract])) OR (Heart Failure, Left-Sided[Title/Abstract])) OR (Myocardial Failure[Title/Abstract])) OR (Diastolic Heart Failure[Title/Abstract])) OR (Heart Failure, Preserved Ejection Fraction[Title/Abstract])) OR (Heart Failure, Normal Ejection Fraction[Title/Abstract])) OR (Heart Failure, Reduced Ejection Fraction [Title/Abstract])) OR (Systolic Heart Failure[Title/Abstract])
#3	(#1) OR (#2)
#4	((Plant Extracts[MeSH Terms]) OR (Phytoestrogens[MeSH Terms])) OR (Drugs, Chinese Herbal[MeSH Terms])
#5	((Plant Extracts[Title/Abstract]) OR (Phytoestrogens[Title/Abstract])) OR (Drugs, Chinese Herbal[Title/Abstract])) OR (Aconitum carmichaelii[Title/Abstract])) OR (Alismatis rhizoma [Title/Abstract])) OR (Allium sativus[Title/Abstract])) OR (Angelica[Title/Abstract])) OR (Angelica sinensis[Title/Abstract])) OR (Astragaloside[Title/Abstract])) OR (Astragaloside IV[Title/Abstract])) OR (Astragalus mongholicus Bunge[Title/Abstract])) OR (Atractylodes[Title/Abstract])) OR (Atractylodes macrocephala[Title/Abstract])) OR (Cannabinoids[Title/Abstract])) OR (Carthamus tinctorius[Title/Abstract])) OR (Cassia twig[Title/Abstract])) OR (Chinese angelica[Title/Abstract])) OR (Cinnamomum cassia[Title/Abstract])) OR (Cinnamon branches[Title/Abstract])) OR (Citri Reticulatae Pericarpium[Title/Abstract])) OR (Codonopsis[Title/Abstract])) OR (Coptis chinensis[Title/Abstract])) OR (Curcumin[Title/Abstract])) OR (Digitalis lanata Ehrh[Title/Abstract])) OR (Dried ginger[Title/Abstract])) OR (Epicarpium citri[Title/Abstract])) OR (Flos Lonicerae[Title/Abstract])) OR (Fructus schisandrae[Title/Abstract])) OR (Garlic-Derived Organic Polysulfides[Title/Abstract])) OR (Ginseng [Title/Abstract])) OR (Ginsenoside[Title/Abstract])) OR (Glycyrrhiza uralensis [Title/Abstract])) OR (Green tea catechins[Title/Abstract])) OR (Hawthorn[Title/Abstract])) OR (Ligusticum chuanxiong[Title/Abstract])) OR (Ligusticum wallichii[Title/Abstract])) OR (Morinda officinalis[Title/Abstract])) OR (Onions[Title/Abstract])) OR (Paeonia lactiflora[Title/Abstract])) OR (Panax ginseng[Title/Abstract])) OR (Panax notoginseng[Title/Abstract])) OR (Poria[Title/Abstract])) OR (Poria cocos[Title/Abstract])) OR (Pueraria montana var. lobata[Title/Abstract])) OR (Radix aconiti carmichaeli[Title/Abstract])) OR (Radix astragali[Title/Abstract])) OR (Radix codonopsis[Title/Abstract])) OR (Radix glycyrrhizae[Title/Abstract])) OR (Radix Ophiopogonis[Title/Abstract])) OR (Radix Praeparata[Title/Abstract])) OR (Red peony[Title/Abstract])) OR (Rhizoma atractylodis macrocephalae[Title/Abstract])) OR (Rhodiola crenulata[Title/Abstract])) OR (Safflower[Title/Abstract])) OR (Salvia miltiorrhiza[Title/Ab

	stract])) OR (Salvianolic acid[Title/Abstract])) OR (Sanchinoside[Title/Abstract])) OR (Semen lepidii[Title/Abstract])) OR (Tangerine peel[Title/Abstract])) OR (Tuckahoe[Title/Abstract])) OR (Vitis vinifera[Title/Abstract])) OR (Wheat Dong[Title/Abstract])) OR (Ze Yuan[Title/Abstract])) OR (Zingiber officinale[Title/Abstract])
#6	(#4) OR (#5)
#7	(#3) AND (#6)

Supplementary Table S2. Search strategy on Embase

#1	heart AND failure OR (heart AND failure, AND diastolic) OR (heart AND failure, AND systolic) OR (cardiac AND failure) OR (heart AND decompensation) OR (heart AND failure, AND congestive) OR (heart AND failure, AND 'right sided') OR (heart AND failure, AND 'left sided') OR (myocardial AND failure) OR (diastolic AND heart AND failure) OR (heart AND failure, AND preserved AND ejection AND fraction) OR (heart AND failure, AND normal AND ejection AND fraction) OR (heart AND failure, AND reduced AND ejection AND fraction) OR (systolic AND heart AND failure)
#2	plant AND extracts OR phytoestrogens OR (drugs, AND chinese AND herbal) OR (aconitum AND carmichaelii) OR (alismatis AND rhizoma) OR (allium AND sativus) OR angelica OR (angelica AND sinensis) OR astragaloside OR (astragaloside AND iv) OR (astragalus AND mongholicus AND bunge) OR atractylodes OR (atractylodes AND macrocephala) OR cannabinoids OR (carthamus AND tinctorius) OR (cassia AND twig) OR (chinese AND angelica) OR (cinnamomum AND cassia) OR (cinnamon AND branches) OR (citri AND reticulatae AND pericarpium) OR codonopsis OR (coptis AND chinensis) OR curcumin OR (digitalis AND lanata AND ehrh) OR (dried AND ginger) OR (epicarpium AND citri) OR (flos AND lonicerae) OR (fructus AND schisandrae) OR ('garlic derived' AND organic AND polysulfides) OR ginseng OR ginsenoside OR (glycyrrhiza AND uralensis) OR (green AND tea AND catechins) OR hawthorn OR (ligusticum AND chuanxiong) OR (ligusticum AND wallichii) OR (morinda AND officinalis) OR onions OR (paeonia AND lactiflora) OR (panax AND ginseng) OR (panax AND notoginseng) OR poria OR (poria AND cocos) OR (pueraria AND montana AND var. AND lobata) OR (radix AND aconiti AND carmichaeli) OR (radix AND astragali) OR (radix AND codonopsis) OR (radix AND glycyrrhizae) OR (radix AND ophiopogonis) OR (radix AND praeparata) OR (red AND peony) OR (rhizoma AND atractylodis AND macrocephalae) OR (rhodiola AND crenulata) OR safflower OR (salvia AND miltiorrhiza) OR (salvianolic AND acid) OR sanchinoside OR (semen AND lepidii) OR (tangerine AND peel) OR tuckahoe OR (vitis AND vinifera) OR (wheat AND dong) OR (ze AND yuan) OR (zingiber AND officinale)

Supplementary Table S3. Search strategy on Cohrane

#1	(Heart Failure) OR (Heart Failure, Diastolic) OR (Heart Failure, Systolic) OR (Cardiac Failure) OR (Heart Decompensation)(systolic AND heart AND failure)
#2	(Heart Failure, Congestive) OR (Heart Failure, Right-Sided) OR (Heart Failure, Left-Sided) OR (Myocardial Failure) OR (Diastolic Heart Failure)
#3	(Heart Failure, Preserved Ejection Fraction) OR (Heart Failure, Normal Ejection Fraction) OR (Heart Failure, Reduced Ejection Fraction) OR (Systolic Heart Failure)
#4	#1 OR #2 OR #3
#5	(Plant Extracts) OR (Phytoestrogens) OR (Drugs, Chinese Herbal) OR (Aconitum carmichaelii) OR (Alismatis rhizoma)
#6	(Allium sativus) OR (Angelica) OR (Angelica sinensis) OR (Astragaloside) OR (Astragaloside IV)
#7	(Astragalus mongholicus Bunge) OR (Atractylodes) OR (Atractylodes macrocephala) OR (Cannabinoids) OR (Carthamus tinctorius)
#8	(Cassia twig) OR (Chinese angelica) OR (Cinnamomum cassia) OR (Cinnamon branches) OR (Citri Reticulatae Pericarpium)
#9	(Codonopsis):ti,ab,kw OR (Coptis chinensis):ti,ab,kw OR (Curcumin):ti,ab,kw OR (Digitalis lanata Ehrh):ti,ab,kw OR (Dried ginger):ti,ab,kw
#10	(Epicarpium citri):ti,ab,kw OR (Flos Lonicerae):ti,ab,kw OR (Fructus schisandrae):ti,ab,kw OR (Garlic-Derived Organic Polysulfides):ti,ab,kw OR (Ginseng):ti,ab,kw
#11	(Ginsenoside):ti,ab,kw OR (Glycyrrhiza uralensis):ti,ab,kw OR (Green tea catechins):ti,ab,kw OR (Hawthorn):ti,ab,kw OR (Ligusticum chuanxiong):ti,ab,kw
#12	(Ligusticum wallichii):ti,ab,kw OR (Morinda officinalis):ti,ab,kw OR (Onions):ti,ab,kw OR (Paeonia lactiflora):ti,ab,kw OR (Panax ginseng):ti,ab,kw
#13	(Panax notoginseng):ti,ab,kw OR (Poria):ti,ab,kw OR (Poria cocos):ti,ab,kw OR (Pueraria montana var. lobata):ti,ab,kw OR (Radix aconiti carmichaeli):ti,ab,kw
#14	(Radix astragali):ti,ab,kw OR (Radix codonopsis):ti,ab,kw OR (Radix glycyrrhizae):ti,ab,kw OR (Radix Ophiopogonis):ti,ab,kw OR (Radix Praeparata):ti,ab,kw
#15	(Red peony):ti,ab,kw OR (Rhizoma atractylodis macrocephalae):ti,ab,kw OR (Rhodiola crenulata):ti,ab,kw OR (Safflower):ti,ab,kw OR (Salvia miltiorrhiza):ti,ab,kw
#16	(Salvianolic acid):ti,ab,kw OR (Sanchinoside):ti,ab,kw OR (Semen

	lepidii):ti,ab,kw OR (Tangerine peel):ti,ab,kw OR (Tuckahoe):ti,ab,kw
#17	(Vitis vinifera):ti,ab,kw OR (Wheat Dong):ti,ab,kw OR (Ze Yuan):ti,ab,kw OR (Zingiber officinale):ti,ab,kw
#18	#5 OR #6 OR #7 OR #8 OR #9 OR #10 OR #11 OR #12 OR #13 OR #14 OR #15 OR #16 OR #17
#19	#4 AND #18

Supplementary Table S4. Search strategy on WOS

#1	(((((((((((((ALL=(Heart Failure)) OR ALL=(Heart Failure, Diastolic)) OR ALL=(Heart Failure, Systolic)) OR ALL=(Cardiac Failure)) OR ALL=(Heart Decompensation)) OR ALL=(Heart Failure, Congestive)) OR ALL=(Heart Failure, Right-Sided)) OR ALL=(Heart Failure, Left-Sided)) OR ALL=(Myocardial Failure)) OR ALL=(Diastolic Heart Failure)) OR ALL=(Heart Failure, Preserved Ejection Fraction)) OR ALL=(Heart Failure, Normal Ejection Fraction)) OR ALL=(Heart Failure, Reduced Ejection Fraction)) OR ALL=(Systolic Heart Failure)
#2	((((((((((((((((((((ALL=(Plant Extracts)) OR ALL=(Phytoestrogens)) OR ALL=(Drugs, Chinese Herbal)) OR ALL=(Aconitum carmichaelii)) OR ALL=(Alismatis rhizoma)) OR ALL=(Allium sativus)) OR ALL=(Angelica)) OR ALL=(Angelica sinensis)) OR ALL=(Astragaloside)) OR ALL=(Astragaloside IV)) OR ALL=(Astragalus mongholicus Bunge)) OR ALL=(Atractylodes)) OR ALL=(Atractylodes macrocephala)) OR ALL=(Cannabinoids)) OR ALL=(Carthamus tinctorius)) OR ALL=(Cassia twig)) OR ALL=(Chinese angelica)) OR ALL=(Cinnamomum cassia)) OR ALL=(Cinnamon branches)) OR ALL=(Citri Reticulatae Pericarpium)) OR ALL=(Codonopsis)
#3	((((((((((((((((((((((ALL=(Coptis chinensis)) OR ALL=(Curcumin)) OR ALL=(Digitalis lanata Ehrh)) OR ALL=(Dried ginger)) OR ALL=(Epicarpium citri)) OR ALL=(Flos Lonicerae)) OR ALL=(Fructus schisandrae)) OR ALL=(Garlic-Derived Organic Polysulfides)) OR ALL=(Ginseng)) OR ALL=(Ginsenoside)) OR ALL=(Glycyrrhiza uralensis)) OR ALL=(Green tea catechins)) OR ALL=(Hawthorn)) OR ALL=(Ligusticum chuanxiong)) OR ALL=(Ligusticum wallichii)) OR ALL=(Morinda officinalis)) OR ALL=(Onions)) OR ALL=(Paeonia lactiflora)) OR ALL=(Panax ginseng)) OR ALL=(Panax notoginseng)) OR ALL=(Poria)) OR ALL=(Poria cocos)) OR ALL=(Pueraria montana var. lobata)
#4	(((((((((((((((((((ALL=(Radix aconiti carmichaeli)) OR ALL=(Radix astragali)) OR ALL=(Radix codonopsis)) OR ALL=(Radix glycyrrhizae)) OR ALL=(Radix Ophiopogonis)) OR ALL=(Radix Praeparata)) OR ALL=(Red peony)) OR ALL=(Rhizoma atractylodis macrocephalae)) OR ALL=(Rhodiola crenulata)) OR ALL=(Safflower)) OR ALL=(Salvia

	miltiorrhiza)) OR ALL=(Salvianolic acid)) OR ALL=(Sanchinoside)) OR ALL=(Semen lepidii)) OR ALL=(Tangerine peel)) OR ALL=(Tuckahoe)) OR ALL=(Vitis vinifera)) OR ALL=(Wheat Dong)) OR ALL=(Ze Yuan)) OR ALL=(Zingiber officinale)
#5	#1 AND #2
#6	#1 AND #3
#7	#1 AND #4

Supplementary Table S5. Consistency test for Number of patients with improved NYHA classification.

Side	Direct Coef.	Std. Err.	Indirect Coef.	Std. Err.	Difference Coef.	Std. Err.	P> |z |
A VS C	-1.145132	0.5517093	-0.2682054	72.81929	-0.8769269	72.82137	0.99
B VS C	-1.009112	0.2565501	-1.281189	281.8162	0.2720769	281.8166	0.999
C VS D	1.144549	0.5523841	3.434847	640.8412	-2.290298	640.841	0.997
C VS E	-0.1658126	0.3869913	2.124308	634.1088	-2.29012	634.1087	0.997
C VS F	0.822491	0.3742115	3.11253	622.6816	-2.290039	622.6815	0.997
C VS G	0.838079	0.1173337	3.128345	325.1942	-2.290266	325.1942	0.994
C VS H	1.540445	0.8705955	3.830626	632.4744	-2.290181	632.4738	0.997
C VS I	0.3166696	0.8008224	2.60668	635.4403	-2.29001	635.4398	0.997
C VS J	0.7672552	1.283881	3.057428	634.6013	-2.290173	634.6	0.997
C VS K	1.94591	0.8280787	4.236097	629.5979	-2.290187	629.5974	0.997
C VS L	1.707676	0.8183428	3.997832	638.6729	-2.290155	638.6724	0.997
C VS M	1.930572	0.8180092	4.220724	446.9662	-2.290152	446.9655	0.996

NOTE: A: Astragalus and codonopsis extract, B: Astragalus extract, C: Conventional treatment, D: Ginkgo biloba extract, E: Hawthorn extract, F: Panax ginseng and Aconite extract, G: Panax ginseng and Ophiopogon japonicus extract, H: Panax ginseng extract, I: Rhodiola extract, J: Red ginseng extract, K: Red ginseng Ophiopogon japonicus and Schisandra extract, L: Salvia miltiorrhiza extract,

M: Water extract of stem bark of Terminalia arjuna.

Supplementary Table S6. League table on Number of patients with improved NYHA classification.

WEOSBOTA	RGOJASE	SME	PGE	AACE,	GBE	AE	RGE	PGAAE	PGAOJE	RE	CT	HE
WEOSBOTA	1.02 (0.10,9.94)	0.80 (0.08,7.73)	0.68 (0.07,7.04)	0.46 (0.07,3.15)	0.46 (0.07,3.15)	0.40 (0.07,2.14)	0.31 (0.02,6.17)	0.33 (0.06,1.93)	0.34 (0.07,1.69)	0.20 (0.02,1.88)	0.15 (0.03,0.72)	0.12 (0.02,0.72)
0.98 (0.10,9.64)	RGOJASE	0.79 (0.08,7.72)	0.67 (0.06,7.02)	0.45 (0.06,3.16)	0.45 (0.06,3.16)	0.39 (0.07,2.14)	0.31 (0.02,6.15)	0.33 (0.05,1.93)	0.33 (0.06,1.70)	0.20 (0.02,1.87)	0.14 (0.03,0.72)	0.12 (0.02,0.73)
1.25 (0.13,12.07)	1.27 (0.13,12.43)	SME	0.85 (0.08,8.80)	0.57 (0.08,3.94)	0.57 (0.08,3.94)	0.50 (0.09,2.67)	0.39 (0.02,7.72)	0.41 (0.07,2.41)	0.42 (0.08,2.12)	0.25 (0.03,2.35)	0.18 (0.04,0.90)	0.15 (0.03,0.91)
1.48 (0.14,15.36)	1.50 (0.14,15.81)	1.18 (0.11,12.29)	PGE	0.67 (0.09,5.08)	0.67 (0.09,5.08)	0.59 (0.10,3.48)	0.46 (0.02,9.65)	0.49 (0.08,3.12)	0.50 (0.09,2.77)	0.29 (0.03,2.99)	0.21 (0.04,1.18)	0.18 (0.03,1.17)
2.19 (0.32,15.17)	2.23 (0.32,15.66)	1.76 (0.25,12.15)	1.48 (0.20,11.19)	AACE,	1.00 (0.22,4.62)	0.87 (0.26,2.88)	0.69 (0.04,10.60)	0.72 (0.20,2.68)	0.74 (0.24,2.22)	0.44 (0.06,2.94)	0.32 (0.11,0.94)	0.27 (0.07,1.01)
2.19 (0.32,15.19)	2.23 (0.32,15.68)	1.76 (0.25,12.16)	1.49 (0.20,11.21)	1.00 (0.22,4.62)	GBE	0.87 (0.26,2.88)	0.69 (0.04,10.61)	0.72 (0.20,2.68)	0.74 (0.24,2.23)	0.44 (0.06,2.94)	0.32 (0.11,0.94)	0.27 (0.07,1.01)
2.51 (0.47,13.49)	2.55 (0.47,13.96)	2.01 (0.37,10.80)	1.70 (0.29,10.08)	1.15 (0.35,3.78)	1.15 (0.35,3.78)	AE	0.79 (0.06,10.22)	0.83 (0.34,2.02)	0.84 (0.48,1.47)	0.50 (0.10,2.60)	0.36 (0.22,0.60)	0.31 (0.12,0.77)
3.20 (0.16,63.25)	3.25 (0.16,64.91)	2.56 (0.13,50.63)	2.17 (0.10,45.31)	1.46 (0.09,22.57)	1.46 (0.09,22.57)	1.27 (0.10,16.58)	RGE	1.06 (0.08,14.53)	1.07 (0.09,13.43)	0.64 (0.03,12.37)	0.46 (0.04,5.75)	0.39 (0.03,5.45)
3.03 (0.52,17.66)	3.08 (0.52,18.26)	2.42 (0.42,14.14)	2.05 (0.32,13.13)	1.38 (0.37,5.10)	1.38 (0.37,5.10)	1.21 (0.50,2.93)	0.95 (0.07,13.01)	PGAAE	1.02 (0.47,2.19)	0.60 (0.11,3.41)	0.44 (0.21,0.91)	0.37 (0.13,1.07)
2.98 (0.59,15.06)	3.03 (0.59,15.60)	2.39 (0.47,12.06)	2.02 (0.36,11.29)	1.36 (0.45,4.11)	1.36 (0.45,4.11)	1.19 (0.68,2.06)	0.93 (0.07,11.66)	0.98 (0.46,2.12)	PGAOJE	0.59 (0.12,2.90)	0.43 (0.34,0.54)	0.37 (0.17,0.81)
5.02 (0.53,47.35)	5.10 (0.53,48.77)	4.02 (0.43,37.91)	3.40 (0.33,34.54)	2.29 (0.34,15.40)	2.29 (0.34,15.40)	2.00 (0.38,10.39)	1.57 (0.08,30.46)	1.66 (0.29,9.38)	1.68 (0.34,8.23)	RE	0.73 (0.15,3.50)	0.62 (0.11,3.53)
**6.89 (1.39,34.26)**	**7.00 (1.38,35.48)**	**5.52 (1.11,27.43)**	4.67 (0.85,25.71)	**3.14 (1.07,9.27)**	**3.14 (1.06,9.27)**	**2.74 (1.66,4.54)**	2.15 (0.17,26.67)	**2.28 (1.09,4.74)**	**2.31 (1.84,2.91)**	1.37 (0.29,6.59)	CT	0.85 (0.40,1.81)
**8.14 (1.38,47.94)**	**8.26 (1.38,49.56)**	**6.51 (1.10,38.39)**	5.51 (0.85,35.64)	3.71 (0.99,13.90)	3.71 (0.99,13.91)	**3.24 (1.30,8.04)**	2.54 (0.18,35.21)	2.69 (0.94,7.72)	**2.73 (1.24,6.03)**	1.62 (0.28,9.26)	1.18 (0.55,2.52)	HE

NOTE: AE：Astragalus extract, AACE：Astragalus and codonopsis extract, CT: Conventional treatment, GBE： Ginkgo biloba extract， HE: Hawthorn extract, PGAAE: Panax ginseng and Aconite extract， PGAOJE: Panax ginseng and Ophiopogon japonicus extract， PGE: Panax ginseng extract， RE： Rhodiola extract, RGE: Red ginseng extract, RGOJASE: Red ginseng Ophiopogon japonicus and Schisandra extract, SME: Salvia miltiorrhiza extract, WEOSBOTA: Water extract of stem bark of Terminalia arjuna.

Supplementary Table S7. Consistency test for LVEF.

Side	Direct Coef.	Std. Err.	Indirect Coef.	Std. Err.	Difference Coef.	Std. Err.	P> |z |

A VS E	-0.7621354	0.5796745	-0.3958621	72.51725	-0.3662733	72.51956	0.996
B VS E	-0.8853266	0.2354521	-0.6390624	257.902	-0.2462642	257.9021	0.999
C VS E	-1.04881	0.4305113	-0.4733169	441.0867	-0.5754926	441.087	0.999
D VS E	-0.1115293	0.5797466	-1.413008	634.6358	1.301479	634.6362	0.998
E VS F	0.2732446	0.5482734	1.796343	635.0123	-1.523099	635.0124	0.998
E VS G	1.584125	0.6322814	3.102277	625.889	-1.518152	625.8891	0.998
E VS H	0.0538843	0.5413085	1.583683	632.7013	-1.529798	632.7015	0.998
E VS I	0.035195	0.3146573	1.559431	366.0308	-1.524236	366.0309	0.997
E VS J	0.3094316	0.5634655	1.889461	632.8366	-1.580029	632.8368	0.998
E VS K	1.622918	0.5759241	3.147153	632.981	-1.524235	632.9811	0.998
E VS K	0.1715482	0.4257587	1.695551	438.8483	-1.524003	438.8484	0.997

NOTE: A: Astragalus and codonopsis extract, B: Astragalus extract, C: Berberine extract, D: Centaurea behen extract, E: Conventional treatment, F: Hawthorn extract, G: Oak wood extract, H: Panax ginseng and Aconite extract, I: Panax ginseng and Ophiopogon japonicus extract, J: Rhodiola extract, K: Salvia miltiorrhiza extract, L: Water extract of stem bark of Terminalia arjuna.

Supplementary Table S8. League table on LVEF.

SME	OWE	BE	AE	AACE	RE	HE	WEOSBOTA	CBE	PGAAE	PGAOJE	CT
SME	-0.04	-0.57	-0.74	-0.86	-1.31	-1.35	**-1.45**	-1.51	**-1.57**	**-1.59**	**-1.62**
	(-1.72,1.64)	(-1.98,0.84)	(-1.96,0.48)	(-2.46,0.74)	(-2.89,0.27)	(-2.91,0.21)	**(-2.86,-0.05)**	(-3.11,0.09)	**(-3.12,-0.02)**	**(-2.87,-0.30)**	**(-2.75,-0.49)**
0.04	OWE	-0.54	-0.70	-0.82	-1.27	-1.31	-1.41	-1.47	-1.53	**-1.55**	**-1.58**
(-1.64,1.72)		(-2.03,0.96)	(-2.02,0.62)	(-2.50,0.86)	(-2.93,0.39)	(-2.95,0.33)	(-2.91,0.08)	(-3.15,0.21)	(-3.16,0.10)	**(-2.93,-0.16)**	**(-2.82,-0.34)**
0.57	0.54	BE	-0.16	-0.29	-0.74	-0.78	-0.88	-0.94	-0.99	-1.01	**-1.05**
(-0.84,1.98)	(-0.96,2.03)		(-1.12,0.80)	(-1.70,1.13)	(-2.13,0.65)	(-2.14,0.59)	(-2.06,0.30)	(-2.35,0.48)	(-2.35,0.36)	(-2.06,0.03)	**(-1.89,-0.21)**
0.74	0.70	0.16	AE	-0.12	-0.58	-0.61	-0.71	-0.77	-0.83	**-0.85**	**-0.89**
(-0.48,1.96)	(-0.62,2.02)	(-0.80,1.12)		(-1.35,1.10)	(-1.77,0.62)	(-1.78,0.56)	(-1.67,0.24)	(-2.00,0.45)	(-1.99,0.33)	**(-1.62,-0.08)**	**(-1.35,-0.42)**
0.86	0.82	0.29	0.12	AACE	-0.45	-0.49	-0.59	-0.65	-0.71	-0.73	-0.76
(-0.74,2.46)	(-0.86,2.50)	(-1.13,1.70)	(-1.10,1.35)		(-2.04,1.13)	(-2.05,1.07)	(-2.00,0.82)	(-2.26,0.96)	(-2.26,0.85)	(-2.02,0.57)	(-1.90,0.37)

1.31	1.27	0.74	0.58	0.45	RE	-0.04	-0.14	-0.20	-0.26	-0.27	-0.31
(-0.27,2.89)	(-0.39,2.93)	(-0.65,2.13)	(-0.62,1.77)	(-1.13,2.04)		(-1.58,1.50)	(-1.52,1.25)	(-1.78,1.39)	(-1.79,1.28)	(-1.54,0.99)	(-1.41,0.79)
1.35	1.31	0.78	0.61	0.49	0.04	HE	-0.10	-0.16	-0.22	-0.24	-0.27
(-0.21,2.91)	(-0.33,2.95)	(-0.59,2.14)	(-0.56,1.78)	(-1.07,2.05)	(-1.50,1.58)		(-1.46,1.26)	(-1.73,1.40)	(-1.73,1.29)	(-1.48,1.00)	(-1.35,0.80)
**1.45**	1.41	0.88	0.71	0.59	0.14	0.10	WEOSBOTA	-0.06	-0.12	-0.14	-0.17
**(0.05,2.86)**	(-0.08,2.91)	(-0.30,2.06)	(-0.24,1.67)	(-0.82,2.00)	(-1.25,1.52)	(-1.26,1.46)		(-1.47,1.35)	(-1.47,1.23)	(-1.17,0.90)	(-1.01,0.66)
1.51	1.47	0.94	0.77	0.65	0.20	0.16	0.06	CBE	-0.06	-0.08	-0.11
(-0.09,3.11)	(-0.21,3.15)	(-0.48,2.35)	(-0.45,2.00)	(-0.96,2.26)	(-1.39,1.78)	(-1.40,1.73)	(-1.35,1.47)		(-1.61,1.50)	(-1.37,1.22)	(-1.25,1.02)
**1.57**	1.53	0.99	0.83	0.71	0.26	0.22	0.12	0.06	PGAAE	-0.02	-0.05
**(0.02,3.12)**	(-0.10,3.16)	(-0.36,2.35)	(-0.33,1.99)	(-0.85,2.26)	(-1.28,1.79)	(-1.29,1.73)	(-1.23,1.47)	(-1.50,1.61)		(-1.25,1.21)	(-1.11,1.01)
1.59	1.55	1.01	0.85	0.73	0.27	0.24	0.14	0.08	0.02	PGAOJE	-0.04
(0.30,2.87)	(0.16,2.93)	(-0.03,2.06)	(0.08,1.62)	(-0.57,2.02)	(-0.99,1.54)	(-1.00,1.48)	(-0.90,1.17)	(-1.22,1.37)	(-1.21,1.25)		(-0.65,0.58)
1.62	1.58	1.05	0.89	0.76	0.31	0.27	0.17	0.11	0.05	0.04	CT
(0.49,2.75)	(0.34,2.82)	(0.21,1.89)	(0.42,1.35)	(-0.37,1.90)	(-0.79,1.41)	(-0.80,1.35)	(-0.66,1.01)	(-1.02,1.25)	(-1.01,1.11)	(-0.58,0.65)	

NOTE: AE:Astragalus extract, AACE: Astragalus and codonopsis extract, BE: Berberine extract, CT: Conventional treatment, CBE: Centaurea behen extract, HE: Hawthorn extract, OWE: Oak wood extract, PGAAE: Panax ginseng and Aconite extract, PGAOJE: Panax ginseng and Ophiopogon japonicus extract, RE: Rhodiola extract, SME: Salvia miltiorrhiza extract, WEOSBOTA: Water extract of stem bark of Terminalia arjuna.

Supplementary Table S9. Consistency test for 6MWT.

Side	Direct Coef.	Std. Err.	Indirect Coef.	Std. Err.	Difference Coef.	Std. Err.	P> |z |
A VS D	-1.093484	0.385494	-0.0784425	119.895	-1.015041	119.8955	0.993
B VS D	-0.4863838	0.4774309	-1.708449	632.4706	1.222066	632.4708	0.998
C VS D	-0.0623623	0.5228227	-2.124545	635.0437	2.062183	635.044	0.997
D VS E	-0.1097058	0.4874731	2.077099	627.2114	-2.186805	627.2116	0.997
D VS F	0.9643812	0.5609822	3.151141	619.1475	-2.186759	619.1476	0.997
D VS G	0.0278363	0.4798874	2.214783	629.1937	-2.186947	629.1938	0.997
D VS H	0.2447794	0.4690579	2.431737	634.9783	-2.186957	634.9784	0.997
D VS I	0.1160187	0.4915097	2.303052	626.3479	-2.187033	626.348	0.997

NOTE: A: Astragalus extract, B: Berberine extract, C: Centaurea behen extract, D: Conventional

treatment, E: Hawthorn extract, F: Oak wood extract, G: Panax ginseng and Aconite extract, H: Panax ginseng and Ophiopogon japonicus extract, I: Water extract of stem bark of Terminalia arjuna.

Supplementary Table S10. League table on 6WMT.

AE	OWE	BE	PGAOJE	WEOSBOTA	CBE	PGAAE	CT	HE
AE	-0.13 (-1.46,1.20)	-0.61 (-1.81,0.60)	-0.85 (-2.04,0.34)	-0.98 (-2.20,0.25)	-1.03 (-2.30,0.24)	-1.07 (-2.27,0.14)	**-1.09 (-1.85,-0.34)**	-1.20 (-2.42,0.01)
0.13 (-1.20,1.46)	OWE	-0.48 (-1.92,0.97)	-0.72 (-2.15,0.71)	-0.85 (-2.31,0.61)	-0.90 (-2.40,0.60)	-0.94 (-2.38,0.51)	-0.96 (-2.06,0.14)	-1.07 (-2.53,0.38)
0.61 (-0.60,1.81)	0.48 (-0.97,1.92)	BE	-0.24 (-1.55,1.07)	-0.37 (-1.71,0.97)	-0.42 (-1.81,0.96)	-0.46 (-1.79,0.87)	-0.49 (-1.42,0.45)	-0.60 (-1.93,0.74)
0.85 (-0.34,2.04)	0.72 (-0.71,2.15)	0.24 (-1.07,1.55)	PGAOJE	-0.13 (-1.46,1.20)	-0.18 (-1.56,1.19)	-0.22 (-1.53,1.10)	-0.24 (-1.16,0.67)	-0.35 (-1.68,0.97)
0.98 (-0.25,2.20)	0.85 (-0.61,2.31)	0.37 (-0.97,1.71)	0.13 (-1.20,1.46)	WEOSBOTA	-0.05 (-1.46,1.35)	-0.09 (-1.43,1.26)	-0.12 (-1.08,0.85)	-0.23 (-1.58,1.13)
1.03 (-0.24,2.30)	0.90 (-0.60,2.40)	0.42 (-0.96,1.81)	0.18 (-1.19,1.56)	0.05 (-1.35,1.46)	CBE	-0.03 (-1.43,1.36)	-0.06 (-1.09,0.96)	-0.17 (-1.57,1.23)
1.07 (-0.14,2.27)	0.94 (-0.51,2.38)	0.46 (-0.87,1.79)	0.22 (-1.10,1.53)	0.09 (-1.26,1.43)	0.03 (-1.36,1.43)	PGAAE	-0.03 (-0.97,0.91)	-0.14 (-1.48,1.20)
**1.09 (0.34,1.85)**	0.96 (-0.14,2.06)	0.49 (-0.45,1.42)	0.24 (-0.67,1.16)	0.12 (-0.85,1.08)	0.06 (-0.96,1.09)	0.03 (-0.91,0.97)	CT	-0.11 (-1.07,0.85)
1.20 (-0.01,2.42)	1.07 (-0.38,2.53)	0.60 (-0.74,1.93)	0.35 (-0.97,1.68)	0.23 (-1.13,1.58)	0.17 (-1.23,1.57)	0.14 (-1.20,1.48)	0.11 (-0.85,1.07)	HE

NOTE: AE: Astragalus extract, BE: Berberine extract, CT: Conventional treatment, CBE: Centaurea behen extract, HE: Hawthorn extract, OWE: Oak wood extract, PGAAE: Panax ginseng and Aconite extract, PGAOJE: Panax ginseng and Ophiopogon japonicus extract, WEOSBOTA: Water extract of stem bark of Terminalia arjuna.

Supplementary Table S11. Consistency test for QoL.

Side	Direct Coef.	Std. Err.	Indirect Coef.	Std. Err.	Difference Coef.	Std. Err.	P> |z |
A VS C	-5.290996	0.3600123	-0.0330272	112.735	-5.257969	112.7355	0.963
B VS C	-0.2644636	0.290696	-10.31776	628.0994	10.0533	628.0996	0.987
C VS D	0.1254504	0.1011463	10.70741	315.7639	-10.58196	315.7639	0.973
C VS E	16.44307	1.986595	27.02497	632.3518	-10.5819	632.3487	0.987
C VS F	0.5800543	0.1794966	11.20068	683.6654	-10.62062	683.6654	0.988
C VS G	-0.0259843	0.228102	10.59533	646.6401	-10.62131	646.6401	0.987

NOTE: A: Berberine extract, B: Centaurea behen extract, C: Conventional treatment, D: Hawthorn extract, E: Oak wood extract, F: Panax ginseng and Ophiopogon japonicus extract, H: Water extract of stem bark of Terminalia arjuna.

Supplementary Table S12. League table on QoL.

OWE	BE	PGAOJE	CBE	HE	WEOSBOTA	CT
OWE	**-11.15 (-15.11,-7.20)**	**-15.86 (-19.77,-11.95)**	**-16.18 (-20.11,-12.24)**	**-16.32 (-20.22,-12.42)**	**-16.47 (-20.39,-12.55)**	**-16.44 (-20.34,-12.55)**
**11.15 (7.20,15.11)**	BE	**-4.71 (-5.50,-3.92)**	**-5.03 (-5.93,-4.12)**	**-5.17 (-5.90,-4.43)**	**-5.32 (-6.15,-4.48)**	**-5.29 (-6.00,-4.59)**
**15.86 (11.95,19.77)**	**4.71 (3.92,5.50)**	PGAOJE	-0.32 (-0.99,0.35)	**-0.45 (-0.86,-0.05)**	**-0.61 (-1.17,-0.04)**	**-0.58 (-0.93,-0.23)**
**16.18 (12.24,20.11)**	**5.03 (4.12,5.93)**	0.32 (-0.35,0.99)	CBE	-0.14 (-0.74,0.46)	-0.29 (-1.01,0.43)	-0.26 (-0.83,0.31)
**16.32 (12.42,20.22)**	**5.17 (4.43,5.90)**	**0.45 (0.05,0.86)**	0.14 (-0.46,0.74)	HE	-0.15 (-0.64,0.34)	-0.13 (-0.32,0.07)
**16.47 (12.55,20.39)**	**5.32 (4.48,6.15)**	**0.61 (0.04,1.17)**	0.29 (-0.43,1.01)	0.15 (-0.34,0.64)	WEOSBOTA	0.03 (-0.42,0.47)
**16.44 (12.55,20.34)**	**5.29 (4.59,6.00)**	**0.58 (0.23,0.93)**	0.26 (-0.31,0.83)	0.13 (-0.07,0.32)	-0.03 (-0.47,0.42)	CT

NOTE: BE: Berberine extract, CT: Conventional treatment, CBE: Centaurea behen extract, HE: Hawthorn extract, OWE: Oak wood extract, PGAOJE: Panax ginseng and Ophiopogon japonicus extract, WEOSBOTA: Water extract of stem bark of Terminalia arjuna.
