# Supplementary material for: Efficacy of plant extracts in heart failure patients: a systematic review and network meta-analysis
Source: BMC Cardiovasc Disord. 2026 Jun 12;26:502. doi: 10.1186/s12872-026-05793-x (PMC13262503; doi:10.1186/s12872-026-05793-x)
Supplement: Supplementary file 2 — Supplementary Material 2. Figure S1. [file 12872_2026_5793_MOESM2_ESM.pdf]

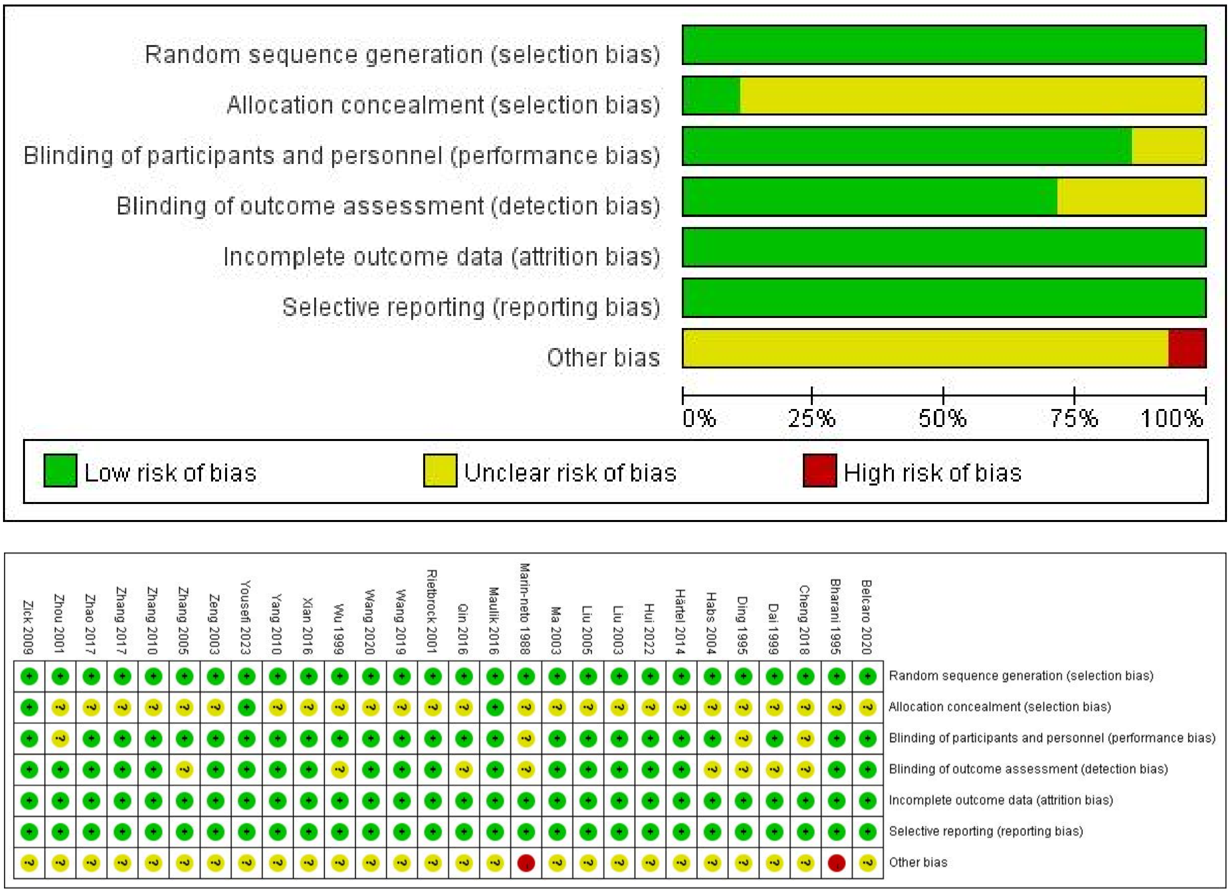

Supplementary Figure S1. Analysis of the risk of bias in accordance with the Cochrane collaboration guideline.
