# Supplementary material for: Efficacy of plant extracts in heart failure patients: a systematic review and network meta-analysis
Source: BMC Cardiovasc Disord. 2026 Jun 12;26:502. doi: 10.1186/s12872-026-05793-x (PMC13262503; doi:10.1186/s12872-026-05793-x)
Supplement: Supplementary file 3 — Supplementary Material 3. File 1. [file 12872_2026_5793_MOESM3_ESM.pdf]

PRISMA 2020 Checklist

Section and Topic	Item #	Checklist item	Location where item is reported
**TITLE**			
Title	1	Identify the report as a systematic review.	Manuscript lines 3, page 1
**ABSTRACT**			
Abstract	2	See the PRISMA 2020 for Abstracts checklist.	Manuscript lines 30-58, page 2-3
**INTRODUCTION**			
Rationale	3	Describe the rationale for the review in the context of existing knowledge.	Manuscript lines 64-82
Objectives	4	Provide an explicit statement of the objective(s) or question(s) the review addresses.	Manuscript lines 114-120
**METHODS**			
Eligibility criteria	5	Specify the inclusion and exclusion criteria for the review and how studies were grouped for the syntheses.	Manuscript lines 140-158
Information sources	6	Specify all databases, registers, websites, organisations, reference lists and other sources searched or consulted to identify studies. Specify the date when each source was last searched or consulted.	Manuscript lines 129-130
Search strategy	7	Present the full search strategies for all databases, registers and websites, including any filters and limits used.	Supplementary Table S1, S2, S3, and S4.
Selection process	8	Specify the methods used to decide whether a study met the inclusion criteria of the review, including how many reviewers screened each record and each report retrieved, whether they worked independently, and if applicable, details of automation tools used in the process.	Manuscript lines 160-168
Data collection process	9	Specify the methods used to collect data from reports, including how many reviewers collected data from each report, whether they worked independently, any processes for obtaining or confirming data from study investigators, and if applicable, details of automation tools used in the process.	Manuscript lines 163-168
Data items	10a	List and define all outcomes for which data were sought. Specify whether all results that were compatible with each outcome domain in each study were sought (e.g. for all measures, time points, analyses), and if not, the methods used to decide which results to collect.	Manuscript lines 133-139
	10b	List and define all other variables for which data were sought (e.g. participant and intervention characteristics, funding sources). Describe any assumptions made about any missing or unclear information.	Manuscript lines 170-173
Study risk of bias assessment	11	Specify the methods used to assess risk of bias in the included studies, including details of the tool(s) used, how many reviewers assessed each study and whether they worked independently, and if applicable, details of automation tools used in the process.	Manuscript lines 175-182
Effect measures	12	Specify for each outcome the effect measure(s) (e.g. risk ratio, mean difference) used in the synthesis or presentation of results.	Manuscript lines 184-188
Synthesis methods	13a	Describe the processes used to decide which studies were eligible for each synthesis (e.g. tabulating the study intervention characteristics and comparing against the planned groups for each synthesis (item #5)).<br><br>Studies were deemed eligible for a specific network meta-analysis (NMA) if they reported data on the corresponding outcome (NYHA improvement, LVEF, 6MWT, or QoL). Consequently, the sets of studies included in each of the four NMAs differed based on data availability.	

Section and Topic	Item #	Checklist item	Location where item is reported
	13b	Describe any methods required to prepare the data for presentation or synthesis, such as handling of missing summary statistics, or data conversions.<br><br>Where necessary, for consistency, standard deviations were imputed from standard errors or confidence intervals reported in the studies. In cases of missing or unclear data, the corresponding authors were not contacted; instead, the study was included only if the necessary data for meta-analysis could be derived from the published report. Disagreements during data extraction were resolved by consensus.	
	13c	Describe any methods used to tabulate or visually display results of individual studies and syntheses.	Manuscript lines 201-211
	13d	Describe any methods used to synthesize results and provide a rationale for the choice(s). If meta-analysis was performed, describe the model(s), method(s) to identify the presence and extent of statistical heterogeneity, and software package(s) used.	Manuscript lines 189-201
	13e	Describe any methods used to explore possible causes of heterogeneity among study results (e.g. subgroup analysis, meta-regression).	NA
	13f	Describe any sensitivity analyses conducted to assess robustness of the synthesized results.	NA
Reporting bias assessment	14	Describe any methods used to assess risk of bias due to missing results in a synthesis (arising from reporting biases).<br><br>The potential for publication bias and small-study effects across the network for each outcome was assessed by visually inspecting the symmetry of comparison-adjusted funnel plots.	
Certainty assessment	15	Describe any methods used to assess certainty (or confidence) in the body of evidence for an outcome.<br><br>The certainty of evidence for the NMA results was not formally assessed using a tool such as GRADE.	
**RESULTS**			
Study selection	16a	Describe the results of the search and selection process, from the number of records identified in the search to the number of studies included in the review, ideally using a flow diagram.	Manuscript Figure 1
	16b	Cite studies that might appear to meet the inclusion criteria, but which were excluded, and explain why they were excluded.	Manuscript lines 214-220
Study characteristics	17	Cite each included study and present its characteristics.	Manuscript Table 1
Risk of bias in studies	18	Present assessments of risk of bias for each included study.	Supplementary Figure S1.
Results of individual studies	19	For all outcomes, present, for each study: (a) summary statistics for each group (where appropriate) and (b) an effect estimate and its precision (e.g. confidence/credible interval), ideally using structured tables or plots.	Supplementary tables (S6, S8, S10, S12)
Results of syntheses	20a	For each synthesis, briefly summarise the characteristics and risk of bias among contributing studies.<br><br>For the four outcome measures (Number of patients with improved NYHA classification, LVEF, 6MWT, QoL), separate network meta-analyses were performed. The analyses included 20, 20, 9, and 9 studies involving 13, 12, 9, and 7 interventions, respectively. The overall risk of bias among contributing studies was generally low, though two studies were noted to have a high risk of bias due to small sample sizes. Consistency tests ($p > 0.05$) indicated acceptable consistency between direct and indirect comparisons for all outcomes.	
	20b	Present results of all statistical syntheses conducted. If meta-analysis was done, present for each the summary estimate and its precision (e.g. confidence/credible interval) and measures of statistical heterogeneity. If comparing groups, describe the direction of the effect.	

Section and Topic	Item #	Checklist item	Location where item is reported
		Bayesian network meta-analyses were conducted for four outcomes. The results are presented as comparisons against conventional treatment (control). The direction of effect favors the plant extract intervention unless otherwise indicated.<br>Improved NYHA class: Several interventions significantly outperformed conventional treatment: Water extract of stem bark of Terminalia arjuna [MD=6.89, 95% CI=(1.39, 34.26)], Red ginseng Ophiopogon japonicus and Schisandra extract [MD=7.00, 95% CI=(1.38, 35.48)], Salvia miltiorrhiza extract [MD=5.52, 95% CI=(1.11, 27.43)], Astragalus and Codonopsis extract [MD=3.14, 95% CI=(1.07, 9.27)], Ginkgo biloba extract [MD=3.14, 95% CI=(1.06, 9.27)], Astragalus extract [MD=2.74, 95% CI=(1.66, 4.54)], Panax ginseng and Aconite extract [MD=2.28, 95% CI=(1.09, 4.74)], Panax ginseng and Ophiopogon japonicus extract [MD=2.31, 95% CI=(1.84, 2.91)]. The SUCRA ranking placed Water extract of stem bark of Terminalia arjuna first (80.4%).<br>LVEF: Interventions superior to conventional treatment included: Salvia miltiorrhiza extract [MD = 1.62, 95% CI = (0.49, 2.75)], Oak wood extract [MD = 1.58, 95% CI = (0.34, 2.82)], Berberine extract [MD = 1.05, 95% CI = (0.21, 1.89)], Astragalus extract [MD = 0.89, 95% CI = (0.42, 1.35)]. Salvia miltiorrhiza extract ranked first (89.7%) based on SUCRA.<br>6MWT: Only Astragalus extract [MD=1.09, 95%CI=(0.34,1.85)] showed superior outcomes compared to conventional treatment. It ranked first (89.5%) based on SUCRA.<br>QoL: Interventions superior to conventional treatment included: Oak wood extract [MD = 16.44, 95% CI = (12.55, 20.34)], Berberine extract [MD = 5.29, 95% CI = (4.59, 6.00)], and Panax ginseng and Ophiopogon japonicus extract [MD = 0.58, 95% CI = (0.23, 0.93)]. Oak wood extract ranked first (100%) based on SUCRA.	
	20c	Present results of all investigations of possible causes of heterogeneity among study results.	NA
	20d	Present results of all sensitivity analyses conducted to assess the robustness of the synthesized results.	NA
Reporting biases	21	Present assessments of risk of bias due to missing results (arising from reporting biases) for each synthesis assessed.	Manuscript Figure 4
Certainty of evidence	22	Present assessments of certainty (or confidence) in the body of evidence for each outcome assessed.	NA
**DISCUSSION**			
Discussion	23a	Provide a general interpretation of the results in the context of other evidence.	Manuscript lines 367-460
	23b	Discuss any limitations of the evidence included in the review.	Manuscript lines 461-485
	23c	Discuss any limitations of the review processes used.	Manuscript lines 461-485
	23d	Discuss implications of the results for practice, policy, and future research.	Manuscript lines 487-496
**OTHER INFORMATION**			
Registration and protocol	24a	Provide registration information for the review, including register name and registration number, or state that the review was not registered.<br><br>This systematic review and network meta-analysis was registered on the PROSPERO international prospective register of systematic reviews, with registration number CRD42024609307.	
	24b	Indicate where the review protocol can be accessed, or state that a protocol was not prepared.<br><br>The study protocol, as part of the registration information, can be accessed via the PROSPERO register using the registration number	

PRISMA 2020 Checklist

Section and Topic	Item #	Checklist item	Location where item is reported
		(CRD42024609307) mentioned above.	
	24c	Describe and explain any amendments to information provided at registration or in the protocol.<br><br>Based on the provided article, no amendments to the registration details or protocol were mentioned.	
Support	25	Describe sources of financial or non-financial support for the review, and the role of the funders or sponsors in the review.	Manuscript lines 502
Competing interests	26	Declare any competing interests of review authors.	Manuscript lines 508
Availability of data, code and other materials	27	Report which of the following are publicly available and where they can be found: template data collection forms; data extracted from included studies; data used for all analyses; analytic code; any other materials used in the review.	Manuscript lines 505-506

From: Page MJ, McKenzie JE, Bossuyt PM, Boutron I, Hoffmann TC, Mulrow CD, et al. The PRISMA 2020 statement: an updated guideline for reporting systematic reviews. BMJ 2021;372:n71. doi: 10.1136/bmj.n71. This work is licensed under CC BY 4.0. To view a copy of this license, visit https://creativecommons.org/licenses/by/4.0/
